# Main Morphological Characteristics of Tubular Polymeric Scaffolds to Promote Peripheral Nerve Regeneration—A Scoping Review

**DOI:** 10.3390/polym13152563

**Published:** 2021-07-31

**Authors:** Josefa Alarcón Apablaza, María Florencia Lezcano, Alex Lopez Marquez, Karina Godoy Sánchez, Gonzalo H. Oporto, Fernando José Dias

**Affiliations:** 1 Research Centre in Dental Sciences (CICO-UFRO), Dental School—Facultad de Odontología, Universidad de La Frontera, Temuco 4780000, Chile; josefa.alarcon@ufrontera.cl (J.A.A.); florencia.lezcano@ufrontera.cl (M.F.L.); gonzalo.oporto@ufrontera.cl (G.H.O.); 2 Program of Master in Dental Science, Dental School, Universidad de La Frontera, Temuco 4780000, Chile; 3 Department of Integral Adults Dentistry, Dental School—Facultad de Odontología, Universidad de La Frontera, Temuco 4780000, Chile; 4 Laboratorio de Cibernética, Departamento de Bioingeniería, Facultad de Ingeniería, Universidad Nacional de Entre Ríos, Oro Verde 3100, Argentina; 5 HAWK—Hochschule für Angewandte Wissenschaften und Kunst, 37085 Göttingen, Germany; alexlopezmarquez@gmail.com; 6 Scientific and Technological Bioresource Nucleus (BIOREN-UFRO), Universidad de La Frontera, Temuco 4780000, Chile; karina.godoy@ufrontera.cl; 7 Center of Molecular Biology and Phamacogenetics, Universidad de La Frontera, Temuco 4780000, Chile

**Keywords:** tissue engineering, polymer, nerve scaffold, morphology, peripheral nerve regeneration, regenerative biology

## Abstract

The “nerve guide conduits” (NGC) used in nerve regeneration must mimic the natural environment for proper cell behavior. Objective: To describe the main morphological characteristics of polymeric NGC to promote nerve regeneration. Methods: A scoping review was performed following the Preferred Reporting Items for Systematic reviews and Meta-Analyses extension for Scoping Reviews (PRISMA-ScR) criteria in the PubMed, Web of Science, Science Direct, and Scientific Electronic Library Online (SciELO) databases. Primary studies that considered/evaluated morphological characteristics of NGC to promote nerve regeneration were included. Result: A total of 704 studies were found, of which 52 were selected. The NGC main morphological characteristics found in the literature were: (I) NGC diameter affects the mechanical properties of the scaffold. (II) Wall thickness of NGC determines the exchange of nutrients, molecules, and neurotrophins between the internal and external environment; and influences the mechanical properties and biodegradation, similarly to NGC (III) porosity, (IV) pore size, and (V) pore distribution. The (VI) alignment of the NGC fibers influences the phenotype of cells involved in nerve regeneration. In addition, the (VII) thickness of the polymeric fiber influences neurite extension and orientation. Conclusions: An NGC should have its diameter adjusted to the nerve with wall thickness, porosity, pore size, and distribution of pores, to favor vascularization, permeability, and exchange of nutrients, and retention of neurotrophic factors, also favoring its mechanical properties and biodegradability.

## 1. Introduction

Peripheral nerve injuries are disabling [1] because the anatomical recovery of damaged peripheral nerves and sensory and motor functions after injury are not ideal [1,2,3]. The axons that sprout and regenerate from the proximal end migrate through the nerve space to effect functional recovery of the injured nerve; however, when the spaces are >3 mm, the regenerating axons lose directionality, leading to loss of permanent function of the nerve, which could even lead to muscle atrophy [4]. As spontaneous regeneration is absent in larger spaces, a mechanical guide is required to direct axonal growth. The literature has described different treatment alternatives, one of which is the autograft that is considered the “Gold Standard” treatment for nerve injuries. Yet, this solution has inherent limitations including donor site morbidity, scar tissue formation, shortage of donor’s nerves, and inadequate return of function [2,3,4,5,6,7,8].

Under this scenario, the “nerve guide conduit” (NGC) or scaffold arose, intending to seek new treatment alternatives for a nerve disruption [9,10]. These must mimic the native environment of the cells grown within them [4,5] and include growth factors or biomolecules that can activate growth and generate necessary signals for cells for regeneration [11,12]. Therefore, knowledge of the anatomy of peripheral nervous tissue provides the foundation on which biomimetic NGCs can develop, featuring not only the tubular construction of the outer wall that emulates the epineurium to guide nerve regeneration [3] but also fibers with extracellular matrix (ECM) biomimetic components [1,13].

Polymeric NGCs have inherent structural characteristics in their manufacture to recreate the function of the injured section. Generally, these scaffolds are constructed of fibers, with sequential layers surrounding an inner space. This inner space, called the lumen, is where the outgrowth, proliferation, and differentiation of the specific cells that participate in nerve regeneration take place [1]. The NGC functions as a guide for axonal growth and separates the internal contents from the surrounding light and medium while allowing selective permeation and transport through the wall. This exchange between the internal and external environment depends on the thickness of the wall, porosity, and pore distribution and size [1,2,12].

Among the materials available for the elaboration of NGC, biocompatible polymers have great interest in the field of nerve regeneration. The term biocompatibility refers to the suitability of a biomaterial that does not cause adverse effects within the organism; i.e., it is an inert, non-toxic material and the organism does not reject it. Biocompatible polymers both synthetic and natural are used to treat, boost or substitute any tissue or organ of the human body as they provide an optimal microenvironment for cell proliferation, migration, and differentiation [1,2,3,4].

An alternative for its preparation is electrospinning [3,4,5]. This is a technique to mimic the structure of the ECM [9] due to the ability to generate polymeric fibers on a micro- or nano-scale, providing a three-dimensional space with more adhesion sites for growth cells [9]. However, the evaluation of the effect of the size and alignment of the fiber is decisive to achieve desirable regenerative results, as it has been shown that they significantly influence the cells that participate in nerve regeneration [3,6,7,10]. On the other hand, numerous studies have shown that porosity and pore size favors the transfer and exchange of nutrients, metabolites, and gases, and in addition, its large surface area improves cell adhesion and growth, which can be spatially controlled [1,2,9,11].

A critical requirement in neural tissue engineering is the choice of an NGC biocompatible, with good mechanical properties, be easily applied, and bioabsorbable [13]. This last one is to avoid the need for a second surgery for its removal and to ensure nerve regeneration at a controllable rate according to axonal growth rates [14], thus ensuring that the canal maintains its shape and protects tissue until functional recovery is achieved [15]. In addition to the properties mentioned, NGC should be designed with specific topographical cues to improve the cellular interaction with the biomaterial and provide the optimal environment for peripheral nerve regeneration [16]. Therefore, morphological characteristics also affect nerve regeneration, and knowledge of the anatomy of the peripheral nerve tissue provides the basis for any development of biomimetic NGC [2,3,4,10].

Accordingly, an ideal NGC should include a combination of optimal size, architecture, and surface properties that result in improved nerve regeneration and subsequent functionality. However, the main morphological characteristics of a scaffold that have not been established that affect nerve regeneration were not found in the literature. Thus, the aim of this literature review is to describe the main morphological characteristics of polymeric NGC that have been proven to influence a supportive environment for cell survival and development, imitating the ECM and normal anatomy, synergistically promoting morphogenesis, differentiation, and homeostasis of nervous tissues [1,17,18,19].

## 2. Methodology

### 2.1. Search Strategy

A scoping review was performed of the main morphological characteristics of a tubular scaffold to promote peripheral nerve regeneration. Our scoping review was performed according to Preferred Reporting Items for Systematic reviews and Meta-Analyses extension for Scoping Reviews (PRISMA-ScR) guidelines [20].

The PubMed, Scopus, and Scientific Electronic Library Online—SciELO, and Web of Science databases were used. The search terms selected were: “Peripheral nerve”, “Scaffold”, “Nanofibrous”, “fibers”, “Tissue-engineered”, “Tissue Guide”, “Tissue Scaffolds”, “Morphology”, “Characteristic”, “Regeneration”, “Nerve Regeneration”, “Neural Growth”, “Polymer”. The keywords were combined with Boolean terms OR and AND. The search was performed between April 2020 and March 2021. In addition, a manual search of the literature was done by reviewing the references in the articles found in the electronic database.

The following search equation was used in PUBMED:

(((((((((characteristic*) OR (“anatomy and histology”[Subheading])) OR (morphometry)) OR (morphology)) OR (morphometrical)) OR (morphological)) AND (((((((((regeneration) OR (“nerve regeneration”)) OR (“peripheral nerve regeneration”)) OR (“Nerve Regeneration”[Mesh])) OR (neural growth)) OR (“neural growth”)) OR (“Neurogenesis”[Mesh])) OR (“Peripheral Nerves”[Mesh])) OR (“Peripheral Nerve Injuries”[Mesh]))) AND ((((((((((scaffold) OR (scaffold nerve regeneration)) OR (tissue engineering)) OR (tissue guide)) OR (regeneration tissue engineering)) OR (“regeneration tissue engineering”)) OR (“Tissue Scaffolds”[Mesh])) OR (polymeric nerve guide duct)) OR (nerve guide duct)) OR (“Nerve guide conduits”))) AND (((((“peripheral nerve”) OR (peripheral nerve injury)) OR (“peripheral nerve injury”)) OR (“Peripheral Nerves”[Mesh])) OR (“Peripheral Nerve Injuries”[Mesh]))) AND ((((((electrospun) OR (electrospun nanofibers)) OR (electrospun fibers)) OR (electrospun nanofiber)) OR (electrospun scaffolds)) OR (“Nanofibers”[Mesh]))

The same search equation was adapted for the other search engines.

### 2.2. Ethical Issues

This literature review is part of the authors’ research projects that will include experimental methodologies in vitro and in vivo (Universidad de La Frontera Ethics Committee Approval Numbers: 091/19 and 074/20). However, for this review, formal ethical approval is not required, as no primary data were collected.

### 2.3. Eligible Criteria

Primary in vitro and animal studies were included where the general objective was to study the influence of one or more morphological characteristics of a tubular polymeric scaffold (NGC) on peripheral nerve regeneration. Full-text articles with no limits on the publication date, written in English or Spanish, were included for the analysis. Articles were excluded that study nerve regeneration in the central nervous system (CNS), that evaluates a biomaterial and not a morphological characteristic, those that describe accessory morphological characteristics not inherent to the morphology of an NGC and non-polymer scaffold construction material.

### 2.4. Article Selection and Data Extraction

Two independent reviewers analyzed articles obtained in the systematic search process by reviewing the titles and abstracts. Articles that fulfilled the eligibility criteria were analyzed in full text to confirm their relevance. In cases of disagreement between the two reviewers, a third reviewer was invited to help resolve the discrepancies of opinion.

The following information was collected from the full-text articles comprising the final selection: authors, publication years, study design, morphological characteristics of tubular polymeric scaffolds, and their influence on the nerve regenerative. The tables used in data extraction were designed by the authors of this review to obtain data relevant to the topic studied.

## 3. Results

### 3.1. Study Selection

**Figure 1:**
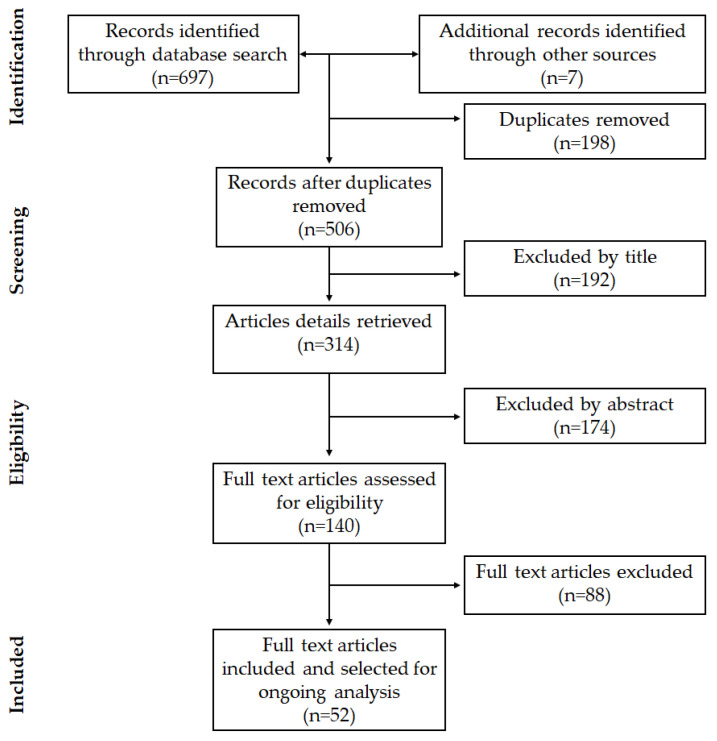
Flow chart for study selection.

The article search and selection process are summarized in Figure 1. The total number of articles found in the databases used was 697, and 7 additional articles were included after the manual search, totaling 704 studies, of which 198 were duplicates.

After the initial reading by title, 192 articles were excluded, of which 41 were systematic reviews, 64 in vitro studies of biomaterials with no regenerative outcomes, 52 studies assessed regeneration of the CNS, and 35 studied the inclusion of adjuvant molecules or characteristics in the NGC.

Among the articles available for abstract evaluation (314 in total), 174 were excluded, of which 7 were literature reviews, 74 were CNS studies, 48 evaluated new scaffold elaboration techniques that did not assess their inherent morphology, and 45 did not evaluate the influence of a morphological characteristic on nerve regeneration.

After reading full-text articles, 88 were excluded, of which 52 did not establish a comparison of measures of morphological characteristics, 13 described a method of scaffold elaboration techniques, 17 did not evaluate nerve regeneration, and 6 were secondary studies.

Finally, in this review, 52 articles were included that corresponded to experimental studies in vitro or in vivo that met the previously defined criteria.

### 3.2. Characteristics of the Selected Studies

The present literature gathered and summarized the evidence in the literature that indicates seven main characteristics that should be considered for the elaboration of an NGC as they are determinants for regenerative success. The characteristics of the polymeric NGC are listed in Table 1, Table 2, Table 3, Table 4 and Table 5.

The polymeric scaffold diameter is a determining factor in the mechanical properties of the tubular scaffold in nerve regeneration [14,21]. Five studies compared in vitro and/or in vivo wall thicknesses in the rat sciatic nerve [13,15,16,22,23]. They determined its influence mainly on the exchange of nutrients, molecules, and growth factors between the internal and external environment. Furthermore, wall thicknesses influence mechanical properties and biodegradation.

Twenty-two in vitro and in vivo studies were included that evaluated the alignment of the scaffold fibers [6,10,41,42,43,44,45,46,47,48,49,50,51,52,53,54,55,56,57,58,59,60]. This characteristic was well reported in the literature that it significantly influences the phenotype of cells involved in nerve regeneration such as Schwann cells (SC), macrophages, and neurons. In addition, the alignment of fibers influences retrograde nerve conduction speed, motor function, and sensory function.

Nine studies evaluated the thickness of the electrospun polymeric fibers [3,5,7,35,36,37,38,39,40], with eight being in vitro and one in vivo. The literature describes the importance of the thickness of these polymeric fibers, mainly in the influence of neurite extension and orientation. Four studies compared the percentage of porosity of scaffolds [24,28,31,34]. Seven evaluated the nerve regeneration in polymeric nerve scaffolds with asymmetric porosity [8,11,25,26,27,29,30], and three studies evaluated the pore size of scaffolds [2,32,33]. This characteristic significantly influences the exchange of nutrients and molecules necessary for nerve regeneration.

## 4. Discussion

### 4.1. Polymeric Nerve Guide Conduits

Various polymers have been used to manufacture artificial nerve canals. Among which we can mention some non-degradable synthetic polymers, such as silicone, biodegradable synthetic polymers, such as poly (lactic-co-glycolic acid (PLGA), poly-ε-caprolactone (PCL), poly-l-lactic acid (PLLA), various natural polymers, such as collagen, chitosan, alginate, elastin, silk, soy protein isolate, fibrin and gelatin and polymers produced by bacterial fermentation e.g., polyhydroxyalkanoates [57,61].

A scaffold must provide structural support to regenerating axons in order to facilitate a higher rate of regeneration [47,48]. Therefore, scaffolds must demonstrate adequate mechanical properties. Natural polymers have decreased mechanical properties and rapid degradation rates, which limits their exclusive use [47,58,60]. However, the presence of synthetic polymers improves these properties [47], which are also favored by the morphological characteristics of the scaffold, such as the alignment of the fibers [48,49,58]. Therefore, the method of mixing synthetic polymers and natural polymers has been widely used with varying degrees of success [47,48,49,54,58]. This mixture improves the bioactivity, biocompatibility, biodegradability of the scaffold, and improves the interaction of the scaffold with the cells [48,58].

In addition, it would be beneficial to mix the synthetic polymers with their hydrophobic structure and lack of sites of cell recognition on the surface [6,48,49,56,58,60] with a natural polymer such as gelatin, collagen, laminin, or fibronectin [35,47,49], as that could promote better cell adhesion [6,30,47,48,57,60], neurite extension [6,47,60], cell proliferation [48,49,57,60], cell differentiation [48], scaffold degradation [48,49,58] and mimicking of their native environment. However, the combination relationship of the two polymers must be evaluated in each situation.

As has been shown in the literature, the composition of the scaffold will directly influence nerve regeneration; however, the morphological characteristics of the NGC are also determining factors for regenerative success. Therefore, this literature review sought and gathered the main morphological characteristics of a polymeric NGC that can influence nerve regeneration. In total, seven main characteristics were identified in the selected studies. The following discussion will address these main aspects relating to nerve regeneration.

### 4.2. Morphological Characteristics of NGCs

#### 4.2.1. Scaffold Diameter/Adjustment

Commercially available NGC is an accepted strategy for overcoming short gaps in nerve repair in which poor clinical results are not uncommon. An unrecognized cause of their failures is the dimension of the canal around the diameter of the nerve [14,21]. Thus, the choice of the diameter of the NGC will depend on the diameter of the injured nerve. The adjustment of the scaffold is an important factor to consider [21]. The implantation of an NGC of small internal diameters can influence the quality of nerve regeneration and maturation and is technically difficult [14,21,22]; faced with this intraoperative dilemma, different sizes of conduits would often have to be chosen [21].

The selection of a large canal technically facilitates implantation to the nerve. However, it would lead to a pronounced collapse of the nerve canal, independent of the polymeric material used, resulting in poor regenerative consequences, muscle atrophy, lower muscle weight, and weak contraction force will occur [21].

On the other hand, a slightly larger channel may represent the most likely type of sizing error in a clinical setting. The NGC size would have the theoretical benefit of being able to accommodate nerve end swelling or scaffold wall swelling and could offer a reasonable fit. However, there are no positive effects compared to better-fitting polymeric tubes [14,21].

**Figure 2:**
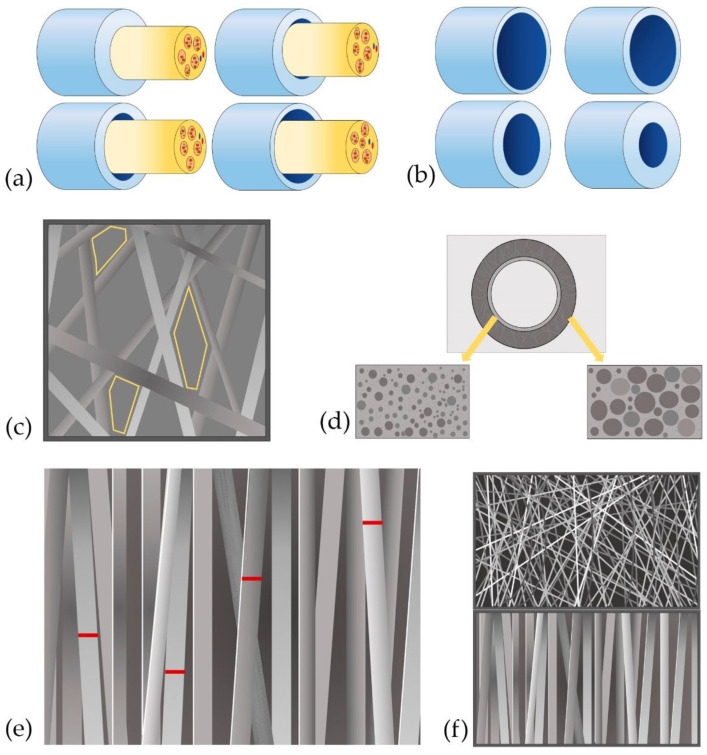
Graphic summary of the main morphological characteristics of NGC. (**a**) NGC diameter (adjustment); (**b**) NGC wall thickness; (**c**) pore size; (**d**) pore distribution; (**e**) thickness of NGC fibers; (**f**) organization of the fibers of the NGC.

The NGC mechanical properties and an eventual collapse of the tubular structure also depend on the scaffold diameter [14,21]. The adjustment of the tubular scaffold could directly influence nerve regeneration, the number, and diameter of regenerating axons and their myelination, as well as subsequent muscle reinnervation (Figure 2) [21].

#### 4.2.2. Scaffold Wall Thickness

Biomolecular signals are required to stimulate cells to regenerate damaged tissue [62]. A promising strategy to improve nerve regeneration obtained with NGC includes the integration of neurotrophic factors (NTFs) [15,23,63,64] as they play an important role in the control of survival, migration, proliferation, and differentiation of cells involved in nerve regeneration [15,65,66] as well as various nutrients important to the cell survival [15].

Increased wall thickness will lead to greater retention of growth factors within the lumen, which may improve the survival capacity of neurons [15,23]. However, these conditions lead to a decrease in the amount of oxygen and the exchange between the internal and external environment of necessary nutrients in the lumen, such as glucose and lysozyme [28,32]. Nevertheless, thinner NGC can be useful for surgical maneuvers [13], but mechanical failures can occur, as revealed in vivo experiments that led to the collapse of the NGC [7,13,32].

Therefore, a suitable NGC wall thickness must provide sufficient mechanical resistance with a minimum thickness that allows its manipulation and the sufficient diffusion of nutrients to ensure that nutrients, molecules, and oxygen reach the preselected Schwann cells (SCs) and regenerate neural tissue while retaining neurotrophic factors [15,16,22,28,32,34].

#### 4.2.3. Porosity of the Scaffold

The architecture of the scaffold affects cellular behavior in nerve regeneration. Therefore, the design and selection of a scaffold with defined porosity will be critical to achieving positive results in nerve repair [8,25]. Porosity is defined as the percentage of void space; it is determined as the ratio of the volume of the pore space divided by the total volume of the object [31].

The superiority of porous NGC has been demonstrated in vivo [8,50,52,55,56,57,58], in vitro [2,11,31,32,33], and pre-experimental studies [27,34]. Different methods of elaboration porous scaffolds have been described in the literature, among which we mainly find; immersion precipitation methods [8,11,27,29,30], electrospinning [2,32], polymer and salt slurry [26,34], electrohydrodynamic jet 3D printing (EHD-jetting) [33], and freeze-drying and freeze-cast molding [31]. The diversity of methods achieves the obtaining of different porous characteristics in the NGC.

The porosity of the scaffold plays a vital role in the exchange of oxygen, nutrients, neurotrophic factors, between the internal and external environment [2,5,11,34], which stimulate and promote cell orientation, infiltration, and migration, in addition to providing a positive influence for axonal growth after nerve injury [5,8,11,24,25,27,28,32,34]. This shows that regenerating axons traverse much longer spaces if the NGC are permeable to the medium [24].

The lack of porosity in the NGC walls decreases and affects nerve regeneration [23,25,30,34]. By contrast, a very porous duct will allow trophic factors to diffuse from the lumen of the NGC to the external environment this loss will prevent the axons from reaching their optimal growth [23]. Therefore, the porosity is a crucial parameter that determines both the diffusion of hydrophilic proteins and the permeability of small molecules such as glucose [28] through the wall to provide an optimal medium for nerve regeneration [28,33].

#### 4.2.4. Pore Size

The pore size of the NGC determines which molecules pass through the graft from the surrounding tissue to the regenerating nerve [25]. Large pores can promote vascularization within NGC [34]; however, if the pores are too large, there is also the risk of cellular infiltration and blockage of the nerve duct by fibrous tissue, providing a less permissive environment for axonal growth, resulting in a low density and number of nerve fibers [26,32,34]. Nevertheless, small pore sizes in the NGC walls decrease and affect nerve regeneration [23,25,30,34]. Therefore, pore size is important in the architecture of a tubular scaffold because it determines the exchange of molecules, growth factors, and nutrients through the wall [24].

A desirable characteristic of an NGC is biodegradability, and this is directly proportional to porosity and pore size [33,67]. Therefore, it is important to consider the size of the pores in different layers of the NGC to obtain desirable results in nerve regeneration.

#### 4.2.5. Distribution and Orientation of Scaffold Pores

Several studies have shown that the interconnected pores of the permeable ducts can increase the exchange of nutrients between the light and the external environment, prevent cellular infiltration that can impede the extension of the axon, and retain neurotrophic factors in the lumen of the NGC [8,26,27]. However, these interconnected pores must be “asymmetrical”, thus the external surface of the NGC should have a larger pore size than the lumen NGC surface.

The method used for the elaboration of NGC with the distribution of polymeric asymmetric pores is the immersion precipitation method. In its basic form, dip precipitation is carried out by dipping a thin film of a concentrated polymer solution into a non-solvent bath. This method achieves asymmetrically porous NGC with selective permeability (to prevent infiltration of fibrous tissue but impregnate nutrients) and hydrophilicity (for effective nutrient permeability) [8,11,27,29,30].

Experimental studies have shown that the NGC requires greater permeability for outflow than for inflow to remove debris, reduce inflammation at the injury site, and accelerate nerve regeneration [8,11,25,26,30]. In addition, these fluidity characteristics allow the exchange of fluids, nutrients, and oxygen, while minimizing fibrous tissue ingrowth, but retain neurotrophic factors, resulting in larger axon diameter and number, a greater number of blood vessels, thicker myelin sheath, faster axonal growth, and SC proliferation [9,45]. Thus, this asymmetry of pore distribution favors these biological events and, consequently, nerve regeneration [8,11,25,29,30,31].

#### 4.2.6. Diameter of Polymer Fiber

A biomimetic NGC should mimic the structure and function of the ECM, defining the optimal architecture to maintain cell organization, viability, invasion, proliferation, and differentiation [39]. The ECM in the peripheral nerves acts as a three-dimensional scaffold consisting of polysaccharide fibers and proteins (collagen fibers, elastin), ranging from tens to hundreds of nanometers [6].

Electrospinning is a promising technique to mimic the structure of the ECM [7,44,45] due to the ability to generate polymeric fibers on a micro- or nano-scale, providing a three-dimensional space with more adhesion sites for growth cells [7,59].

Understanding the influence of micro and nanoscale topography on cell behavior is crucial for the design of functional scaffolds for nerve tissue engineering [29]. Different studies have reported that the polymeric fiber diameter of NGC could alter cell differentiation, morphology, growth, proliferation, and migration [3,5,7,35,36,37,38,39,46,50,52,53,54,55,56,58,59,60]. Furthermore, the average diameter of electrospun fibers in scaffolds is a control variable, useful for manipulating the release profile of growth factors [40]. Therefore, the polymeric fiber diameter is of great importance to promoting nerve regeneration, and this is achieved by varying different electrospinning parameters, such as concentration of the polymer, flow rate, voltage, distance, and collector speed [7,35,36,39]. Among the variables of the polymeric solution, it was shown that the diameter of the fiber is directly proportional to the polymer concentration [35,38].

The diameter of the polymer fiber also influences the mechanical properties of fibrous scaffolds influencing the ultimate tensile strength (UTS), elastic modulus (EM), and elongation at break (EB) [40]. There is evidence that cells respond to a natural-like environment. Similarly, natural characteristics could give cells physical clues that favor the differentiation of stem cells and the growth of neurites [7].

#### 4.2.7. Alignment of the Polymer Fibers

During nerve regeneration, axon growth and neurite outgrowth occur in response to chemical and physical signals derived from the local microenvironment [45]. Therefore, the topography of the fibers within the designed scaffold plays a crucial role in mimicking the ECM [47,48]. The superiority of an electrospun fibrous NGC architecture was demonstrated, which can be conveniently arranged to provide better topographic cues for cells in nerve regeneration [6,7,37,40,42,44]. The native ECM has a specific architecture, which is important for tissue function. Therefore, following a nerve injury, a well-defined architecture is necessary to mimic the ECM accurately to guide nerve regeneration. This is the reason the orientation of polymers fibers of NGC is crucial to promoting nerve regeneration [42,44].

Macrophages are recruited within a few hours to the nerve injury site and constitute an important cellular component in peripheral nerve regeneration by removing myelin debris and secreting numerous bioactive cytokines that play critical roles in regulating nerve regeneration [59]. The polymeric nanofiber arrangement differentially regulates the polarization and pro-curative or pro-inflammatory phenotype of macrophages, and this subsequently influences the outcome of nerve regeneration [59]. The distribution of the fibers also influenced the adhesion, proliferation, survival, differentiation, migration, and growth of different types of cells that participate in peripheral nerve regeneration [35,37,41,43,45,46,48,49,50,51,52,53,54,55,56,57,59,60,68].

In addition, the orientation of the polymeric fibers in the NGC determines the neurite phenotype [35,45,46,48,49,55,60], influencing the proliferation, neurite outgrowth, cell differentiation [55,68] and the sensory [69] and motor [47,50,56,69] nerve regeneration speed, significantly determining functional recovery [69].

### 4.3. Biodegradable Properties

**Figure 3:**
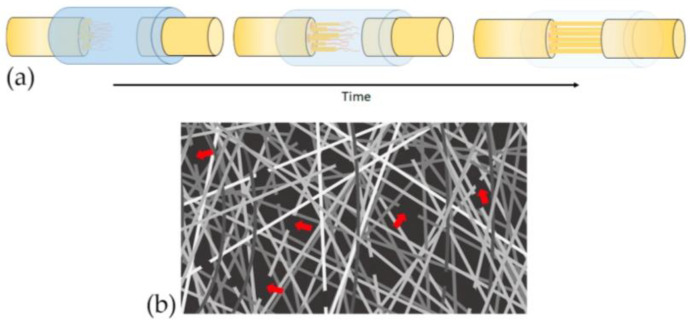
Biodegradability of polymeric scaffolds over time: (**a**) represents scaffold biodegradation at a controllable rate according to axonal growth rates towards the distal nerve site; (**b**) fiber degradation is indicated by the arrows.

A successful nerve guide must comply with established parameters of biodegradation, because bio-durable NGC has the disadvantage of remaining in the body, causing an inflammatory response, and consequently a second intervention may be necessary for its extraction, which can cause an injury to the regenerated nerve [14]. In contrast, NGCs that degrade very rapidly do not provide optimal guidance in the required time. Figure 3a,b represent scaffold biodegradation at a controllable rate to avoid the need for a second surgery, and to ensure nerve regeneration at a controllable rate according to axonal growth rates towards the distal stump [14], guaranteeing that the canal maintains its shape and protects the tissue until functional recovery is achieved [15].

Biodegradability is mainly determined by the polymeric material. Non-degradable polymers have been widely used for the study of nerve regeneration due to their inert and mechanical properties. However, non-degradable polymers may induce chronic inflammatory response and pain by nerve compression because they remain in situ without degradation, which could require a reoperative surgery for the conduit removal. Recent researches for NGC fabrications have concentrated on biodegradable synthetic polymers, including polylactic acid (PLA), poly (glycolic acid) (PGA), poly (lactic-co-glycolic acid) (PLGA), and poly (phosphoester) [27,48]. One of the most effective methods to develop new scaffolds is the alloy of synthetic and natural polymers that can promote nerve regeneration as natural polymers improve the hydrophilicity of composite scaffolds and increase the rate of degradation [31,48,58].

Various studies have shown that the biodegradable property of NGC will also be influenced by the morphological characteristics of the scaffold [31,33,40,67]. Table 6 represents the behavior of the biodegradability of an NGC with the different morphological characteristics. The overall results indicated that biodegradability is inversely proportional to fiber diameter [40] and is directly proportional to porosity and pore size, where the higher the pore size and porosity, the high the degradation rate [31,33,67], due to auto-catalyzed degradation as larger pore sizes allow more PBS solution to be contained per unit volume of scaffolds [33]. On the other hand, the orientation of the fiber plays a major role in the tailoring of the scaffold properties such as degradability. The degradation was greater in random fibers compared to aligned fibers [44]. Therefore, it is important to consider the morphological characteristics of the NGC to obtain desirable results in nerve regeneration. As gradual biodegradation is desired at a controllable rate according to axonal growth rates to ensure that the canal maintains its shape and protects the tissue until functional recovery is achieved [15,33,48] as the mechanical properties deteriorated with the degradation of NGC [33,43].

### 4.4. Mechanical Properties

The type of polymeric material plays a significant role in the mechanical properties of the scaffold [27,34]. However, the morphological characteristics of NGC are determinants in the mechanical properties of the tubular scaffold, to create an environment conducive to nerve regeneration.

**Figure 4:**
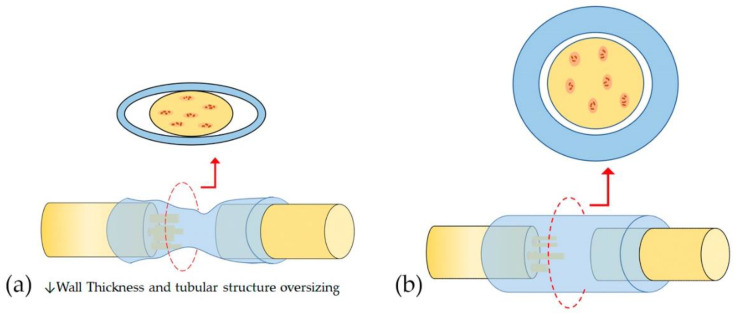
The behavior of the wall thickness and scaffold adjustment in the collapse of the NGC. (**a**) Shows imperfect filling and typical collapse of the NGC produced by low (↓) wall thickhness and tubular structure oversizing and with low wall thickness; (**b**) shows the behavior of an NGC adjusted to the peripheral nerve with an adequate wall thickness, achieving maintaining its tubular construct and exhibiting complete filling of the lumen.

Controlling the fit of the tubular scaffold will protect regenerating axons from compression owing to the collapse of the tubular structure oversizing [21]. Similarly, the wall thickness directly influences this mechanical resistance, important to maintain a stable support structure for nerve regeneration [27]. A very thin wall thickness collapses without additional force (Figure 4a). It is well known that with the increase in conduit wall thickness, the strength of the conduit to resist compression increases accordingly [32]. However, the exchange of nutrients and oxygen between light and the external environment decreases with the increasing thickness of the conduit wall and the increased rigidity is a factor for the failure of the conduit [16]. The behavior of the wall thickness and the tubular scaffold adjustment in the collapse of the NGC is graphically represented in Figure 4. Figure 4a shows a typical collapse of the NGC oversized with imperfect adjustment. Oppositely, Figure 4b shows the behavior of an NGC adjusted to the peripheral nerve with an adequate wall thickness, managing to maintain its tubular construction and exhibiting a complete adjustment of the lumen. Therefore, a minimum wall thickness is required that allows the exchange of molecules necessary for nerve regeneration but that provides balanced mechanical resistance for the neural scaffold to resist pressure from handling, suturing, and surrounding tissue [2,16].

**Figure 5:**
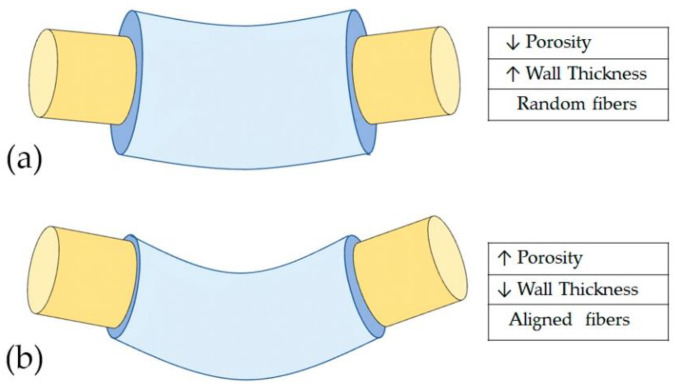
Schematic diagram of the bending stiffness of NGC according to the morphologies characteristic. (**a**) Lower porosity, higher wall thickness, and random fibers provide higher bending stiffness. (**b**) Higher porosity, lower wall thickness, and aligned electrospun fibers provide significantly lower bending stiffness.

The mechanical properties of NGC should provide sufficient resistance to tolerate surgical procedures, subsequent tissue movements associated with patient movement, especially when tissue begins to infiltrate through scaffolds and axonal extension occur [29,31,58]. These mechanical properties, such as Young’s modulus, yield stress, yield strain, ultimate stress, and ultimate strain, are determined by the morphological characteristics of NGC [33]. Mechanical properties were inversely proportional to pore size and porosity [33]. On the other hand, the bending stiffness was significantly affected by the porosity [34] and wall thickness [16]. Figure 5 shows the behavior of the bending stiffness of NGC according to morphological characteristics. The higher the porosity and the lower the wall thickness, the bending stiffness is significantly lower [16,34] (Figure 5b). Consequently, the greater the wall thickness the flexural rigidity is significantly higher [16] (Figure 5a). As is known, a thin wall thickness and higher porosity and pore size facilitate the better mass transfer of nutrients, growth factors, and oxygen. However, mechanical properties are compromised as porosity increases and wall thickness decreases, therefore there must be a balance between facilitating nutrient exchange and sufficient mechanical properties to maintain the tubular structure until nerve regeneration [33].

**Figure 6:**
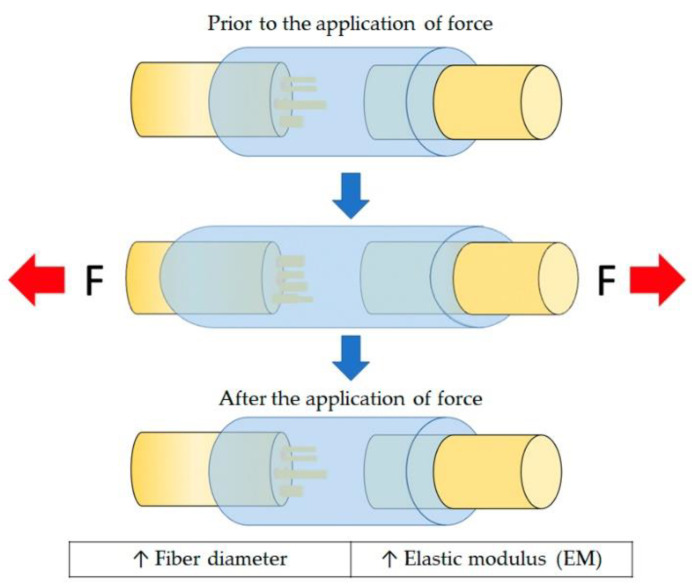
Schematic diagram of the modulus of elasticity (EM) of NGC. The direction of the horizontal arrows indicates the orientation of the applied force. The diameter of the fiber is directly proportional to the elastic modulus of the NGC.

Moreover, the distribution and thickness of the fiber also directly influence the mechanical properties of NGC. Coarse fibers have significantly more elastic modulus [49]. Figure 6 represents the elastic modulus of an NGC. The diameter of the fiber is directly proportional to the elastic modulus of the NGC. Thus, the greater the thickness of the fiber, the greater the elastic modulus, therefore the more rigid will be the NGC.

**Figure 7:**
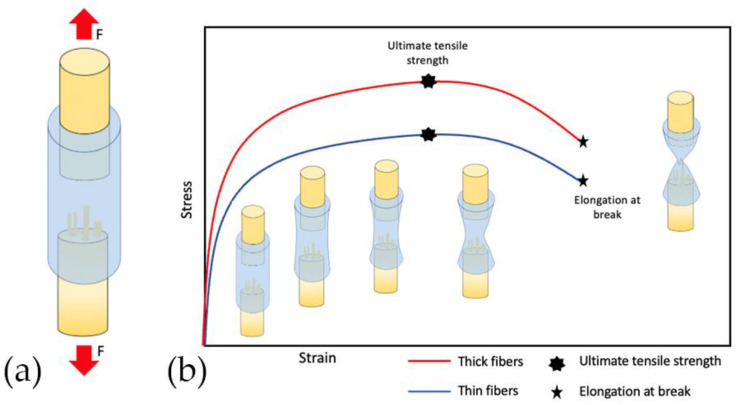
The schematic diagram for the stress–strain curve of NGC. (**a**) The arrows indicate the orientation of the applied force. (**b**) The uphill slope represents the increase in traction in the NGC. Ultimate tensile strength is the maximum in the curve, which means the maximum tension that the NGC can sustain in tension. The downward slope of the curve after UTS is caused by the instability caused by the reduction of the cross-section, which will lead to the break of NGC represented with EB. Coarse fibers have significantly higher ultimate tensile strength (UTS) and elongation at break (EB).

Furthermore, thick fibers have significantly more ultimate tensile strength (UTS) and elongation at break (EB) [40]. Figure 7 shows a schematic diagram for the stress–strain curve of NGC that comparing thin and thick fibers. The strength of an NGC considers the relationship between the external loads applied to materials (Figure 7a) and the resulting deformation or change in material dimensions. In Figure 7b the uphill slope represents the increase in traction in the NGC. Ultimate tensile strength is the maximum in the curve, which means the maximum tension that the NGC can sustain in tension. Thin fibers have a lower maximum tensile strength than thick fibers. The downward slope of the curve after UTS is caused by the instability ty caused by the reduction of the cross-section, which will lead to the break of NGC represented with EB. NGC with thin fibers has significantly less elongation at break (EB) than thick fibers. Therefore, increasing the fiber diameter could significantly improve the mechanical properties of fibrous scaffolds [40].

The mechanical properties provided by the randomly oriented and aligned electrospun fibers differed considerably in all studies [43,44,48,58]. The flexibility and elongation for the aligned electrospun fibers were all greater than those for the random fibers (Figure 5a,b) [44], however, Young’s modulus and tensile strength are controversial in the literature [44,49,58]. Aligned fibers are significantly favorable for nerve regeneration, however, despite the good flexibility and elongation properties of aligned NGCs that allow movement in the injured area, it is difficult to use them for surgical applications due to their insufficient mechanical resistance, independent of the polymeric material, due to the fewer contact points for the aligned nanofibers [2,43]. Several studies have studied the use of a bilayer NGC of aligned nanofibers on the luminal surface, capable of improving the proliferation, migration, and differentiation of neuronal cells and random nanofibers on the external surface to improve mechanical properties [2,51].

### 4.5. Limitations of the Study

The present literature review aimed to obtain, organize and understand the existing evidence in the literature on the morphological characteristics of NGC that would favor nerve regeneration. Although we were systematic in our review, we may have missed publications due to the large number of studies evaluating NGC for nerve regeneration. However, we believe that this was minimized due to the sensitive search strategy used, the additional manual search of the literature, and the double-independent review process used. In addition, the evidence described in this study gives a clear statement of the importance of the seven main morphological characteristics of tubular scaffold to promote peripheral nerve regeneration; therefore, the loss of any publication will not significantly alter what is described in this study. Furthermore, the authors understand that this review fulfills the function of being a first step in the organization and understanding of the evidence in the literature; this scoping review could serve as a basis for the development of future systematic reviews on the same topic.

## 5. Conclusions

Currently, advances in tissue engineering allow the development of a wide range of intelligent polymeric materials as candidates for the development of nerve guide channels. One challenge is making the NGC design better meet the needs of the affected tissue. This review may be crucial to understanding the main morphological characteristics of the NGC to successfully promote nerve regeneration. A morphologically ideal NGC should have its diameter adjusted to the nerve in which it will be implanted and present a wall thickness, porosity, pore size, and asymmetric distribution of pores, to favor vascularization, permeability, and exchange of nutrients and retention of neurotrophic factors. Thus, avoiding undesirable cell infiltration and always considering the need to maintain biomechanical properties and biodegradability. In polymeric NGC prepared by electrospinning, the thickness of the polymeric fibers on a nanoscale and micrometric scale as well as the alignment of these fibers suggest favoring peripheral nerve regeneration. This scaffold design is challenging given the need to create a supporting structure for cells to proliferate and grow, without affecting the overall function of the resulting structure. In the future, more in vitro and in vivo controlled studies are necessary that incorporate the morphological characteristics discussed in this review to confirm how to favor nerve regeneration. This characterized NGC model will help to reduce the number of in vitro and in vivo experimental studies, as it will provide the initial morphological parameters in its elaboration ensuring regenerative success.

## Figures and Tables

**Table 1:** Studies evaluating the NGC diameter (adjustment).

Study	Type of Study	NGC Material	Morphological Parameter	Main Outcome
Den Dunnen et al., 1995 [14]	In vivo Sciatic nerve/rats	Lactic acid-caprolactone	Tube dimensions (Diameters) NGC Wall thicknesses	NGC diameters influenced biodegradation, foreign body reaction, and nerve regeneration.
Isaacs et al., 2014 [21]	In vivo Sciatic nerve/female Sprague-Dawley (SD) rats	Collagen (type I)	Tube dimensions (Diameters)	NGC diameters influenced tube collapse, nerve regeneration, and decreased muscle reinnervation.

**Table 2:** Studies evaluating the NGC Wall thicknesses.

Study	Type of Study	Material	Morphological Parameter	Main Outcome
Perego et al., 1994 [13]	In vitro	Poly(l-lactide-*co*-6-caprolactone)	Wall thicknesses	Wall thicknesses influenced values of tensile strength, percent elongation at break, and elastic modulus.
Den Dunnen et al., 1998 [22]	In vivo Sciatic nerve/rat	Copolymer of DLlactide and e-caprolacone	Internal diameter Wall thicknesses	Evaluated the biodegradation, collapsed, nerve regeneration.
Rutkowski et al., 2002 [15]	In vitro: Schwann Cell (SC) Cultures. Dorsal root ganglia (DRG) of SD rats.	Poly-d, l-lactide	The computer model predicts the wall thickness, porosity, and SC seeding density needed to maximize the axon extension rate while ensuring that sufficient nutrients to the neurons.	Transport of nutrients, nerve growth factor, oxygen, glucose, and nerve regeneration.
Rutkowski et al., 2002 [23]	In vitro: SC Cultures. DRG of SD rats.	Poly-d, l-lactide with SC	Porosity Wall thicknesses	Wall thickness and porosities influenced the number of nutrients and growth factors made available to the neural tissue. Higher porosities: more growth factors diffused out of the conduit. Low porosities: competition for nutrients.
Mobasseri et al., 2015 [16]	In vitro: stem cells differentiated to Schwann cell-like cells. In vivo: SD rat sciatic nerve injury.	Poly ε-caprolactone (PCL) and polylactic acid (PLA)	Wall thicknesses	Increasing the thickness of the wall increased stiffness and limited the permeability of the canal, so it did not show any positive effect on the biological response of the regenerating nerve.

**Table 3:** Studies evaluating porosity, size and distribution of pores of NGC.

Study	Type of Study	Material	Fabrication Technique	Morphological Parameter	Main Outcome
Jenq et al., 1987 [24]	In vivo: sciatic nerve of SD rats	Silicone	Not reported	Porosity	The regenerating axons crossed much longer gaps if the tube was permeable.
Chang et al., 2006 [25]	In vivo: sciatic nerve defects in SD rats.	Poly(dl-lactic acid-*co*-glycolic acid) (PLGA)	Immersion precipitation method	Conduits with asymmetric porosity (The external surface of the NGC has a larger pore size than the lumen surface).	Asymmetric PLGA NGC showed a stable supporting structure, inhibiting exogenous cell invasion during the regeneration, higher regenerated axons at the mid-conduit, and distal nerve site. Asymmetric structure in the NGC wall enhanced the removal of the blockage of the waste drain from the inner inflamed wound in the early stage.
Vleggeert-Lankamp et al., 2007 [26]	In vivo: sciatic nerve of Wistar rat	Poly-(ε-caprolactone)	NaCl as porosifying agent preparing porous structures	Size of pores. Conduits with asymmetric porosity.	The pore size of the outer and inner layers influenced tissue bridge formation, myelination, nerve regeneration, electrophysiological response rate, and muscle reinnervation.
Oh et al., 2007 [27]	Pre-Experimental Study of Biomaterials Development (BD)	Poly(lactic-*co*-glycolic acid) PLGA and Pluronic F127	Modified immersion precipitation	Conduits with asymmetric porosity—nanopores on the inner surface and micropores on the outer surface.	Asymmetric NGC influenced optimal mechanical properties and hydrophilicity and affected nutrient permeability.
Chan et al., 2007 [11]	In vitro: SC and FibroblastsIn vivo: sciatic nerve of SD rats	Poly(DL-lactic acid-*co*-glycolic acid) (PLGA)	Immersion–precipitation phase inversion using a casting process	Asymmetric poreswith high, medium, and low porosity.	Asymmetric porosity NGC influenced the nutrients and oxygen permeation, and proliferation of SC. Also prevented fibrous scar tissue invasion. Unidirectional permeability NGC showed more myelin fibers than the high bidirectional patency NGC.
Oh et al., 2008 [8]	In vivo: Sciatic nerve of SD rats	Poly(lactic-co-glycolic acid) (PLGA) and Pluronic F127	Modified immersion precipitation method	Conduits with asymmetric porosity.	Asymmetric porosity NGC influenced the infiltration of fibrous tissue, neurotrophic factors, and nutrients. Allowing vascular growth for effective delivery of nutrients and oxygen, resulting in rapid and continuous axonal growth.
Kokai et al., 2009 [28]	Pre-Experimental Study of BD	Poli (caprolactona) (PCL)	Dip-coating/salt-leaching technique	Wall thickness Porosity Pore size	The wall thickness influences permeability molecules as lysozyme and glucose. The porosity determines the interconnected through-pores for transluminal flow and solute diffusion.
Oh et al., 2013 [29]	In vivo: Sciatic nerve of rats	Polycaprolactone (PCL)/Pluronic F127	Immersion precipitation method	Asymmetric pores	Nerve fibers regenerated along the longitudinal direction through the NGC with a nano-porous inner surface, while they were grown toward the porous wall of the NGC with a micro-porous inner surface.
Choi et al., 2014 [30]	In vivo: Recurrent laryngeal nerve of rabbits	Polycaprolactone (PCL)/Pluronic F127	Immersion precipitation method	Conduits with asymmetric porosity	Asymmetrically porous PCL/F127 NGC tubes facilitated nerve regeneration compared with nonporous silicone tubes.
Kim et al., 2016 [2]	In vitro: PC12 and S42 cells	Poly lactic-co-glycolic acid (PLGA) and polyurethane (PU)	Electrospinning	Alignment of fibers Pore size	The alignment of fibers affects the porosity and pores diameter. The pore diameter in the aligned nanofibrous mat was 3× larger and the porosity of the aligned nanofibrous scaffolds was higher. Additionally, alignment nanofibers influence cells proliferation and migration
Ghorbani et al., 2017 [31]	In vitro: L929 fibroblast cells	Poly (lactic-*co*-glycolic acid) (PLGA)	Freeze-drying and freeze-cast molding method	Porosity Pore size	Randomly oriented pore (freeze-dried) and interconnected pore (freeze-cast) scaffolds mimic ECM to support cellular adhesion and migration. Different scaffold manufacturing processes affect their properties by altering the microstructure of pores.
Huang et al., 2018 [32]	In vitro: DRG cells cultures In vivo: not reported	Poly(ε-caprolactone) (PCL) sheaths and collagen-chitosan (O-CCH) filler.	Electrospinning	Pore size Wall thickness	NGC pore size influenced the fibroblast invasion and the mechanical strength. Porosity influences nerve regeneration and functional recovery.
Vijayavenkataraman et al., 2018 [33]	In vitro: PC12 cells	Poli (ε-caprolactona) (PCL)	Electrohydrodynamic jet 3D printing (EHD-jetting)	Pore size Porosities	Pore size and porosity significantly influenced mechanical properties and scaffold degradation.
Pawelec et al., 2019 [34]	Pre-Experimental Study of BD	Poly(lactide co-glycolide) (PLGA) Poly(caprolactone) (PCL)	Polymer + salt slurry	Porosity Wall thickness	Porosity and biomaterials influenced the compliance and bending stiffness. Porous PLGA scaffolds were stiffer than porous PCL, fracturing in bend tests. PCL showed high compliance without signs of deformation/kinking after bending.

**Table 4:** Studies evaluating diameter of fibers of NGC.

Study	Study Type	Material	Fabrication Technique	Morphological Parameter	Main Outcome
Yang et al., 2005 [35]	In vitro: Neural stem cells (NSCs)	Poly(l-lactic acid) (PLLA)	Electrospinning	Aligned fibers and diameters of scaffold fibers	The alignment of the scaffold fibers influenced cell orientation. SC differentiation rate was affected by the diameter of the scaffold fiber.
Wen et al., 2006 [36]	In vitro: DRG explants	Poly(acrylonitrile-*co*-vinyl chloride) (PAN-PVC)	Wet-phase inversion process	Diameters of scaffold fibers	The diameter of scaffold fibers influenced alignment, the outgrowth of neurite, and SC migration.
Wang et al., 2010 [37]	In vitro: DRG	Poly-l-lactic acid (PLLA)	Electrospinning	Diameters of scaffold fibers in aligned fibers	Diameter of scaffold fibers influenced neurite extension and SC migration.
Daud et al., 2012 [38]	In vitro: I. neuronal or primary SC cultures alone; II. neuronal and primary SC in co-culture; III. isolated DRG cultures, containing both neuronal and SC.	Polycaprolatone	Electrospinning	Diameters of scaffold fibers	The diameter of scaffold fibers influenced neurite extension or SC migration.
Jiang et al., 2014 [3]	In vivo: adult female SD rats	Poly (ε-caprolactone) (PCL)	Electrospinning	Diameter scaffold of fibers	The diameter of the scaffold fibers affected the number of recovered myelinated axons and the thickness of myelin sheaths. Nanofiber conduits possessed a smaller pore size compared to microfiber conduits.
Gnavi et al., 2015 [39]	In vitro: Explant cultures of SC and DRG Ex vivo: SC	Gelatin	Electrospinning	Diameter of scaffold fibers	The diameters of fibers: influenced SC migration, proliferation and adhesion, and axonal outgrowth; affected actin cytoskeleton organization; and influenced migration rate, motility, and axonal density.
Hu et al., 2016 [7]	In vitro: PC12 cells	Poly(ε-caprolactone) (PCL)-Nerve Growth Factor (NGF) and Bovine Serum Albumin (BSA)	Emulsion electrospinning technique	Aligned fibers and diameters of scaffold fibers	Aligned scaffold nanofibers presented similar diameters to randomly aligned nanofibers, but the aligned nanofibers were uniform. The diameter of fibers influences the neurite length.
Liu et al., 2018 [40]	In vitro: Rat adrenal pheochromocytoma (PC12) cell line	poly(d, l-lactic acid) (PDLLA) and poly(lactic-*co*-glycolic acid) (PLGA)	Dual-source dual-power electrospinning (DSDP-ES)	Diameter of scaffold fibers	The diameter of scaffold fibers influences the ultimate tensile strength, elastic modulus, and elongation at break.
Lizarraga et al., 2019 [5]	In vitro: NG108-15 neuronal cells and Schwann cells	Poly(3-hydroxybutyrate) P(3HB)poly(3-hydroxyoctanoate) P(3HO) 25:75 % P(3HO)/P(3HB) blend (PHA blend)	Electrospinning	Diameter of scaffold fibers	A direct correlation between fiber diameter and neuronal growth and differentiation was noted.

**Table 5:** Studies evaluating alignment of the NGC polymeric fibers.

Study	Type of Study	Material	Fabrication Technique	Main Outcome
Yao et al., 2009 [41]	In vitro: PC12 cells	Polycaprolactone (PCL)	Electrospinning	Aligned scaffold fibers influenced cell orientation and elongation independent of fiber diameter.
Koh et al., 2010 [42]	In vivo: Sciatic nerve of Wistar rats	Poly(l-lactic acid) (PLLA) and laminin–PLLA	Electrospinning	Alignment of the scaffold fibers influenced neuronal growth through injury, and sensory and motor functional recoveries.
Zhu et al., 2011 [43]	Tensile testing and In vitro degradation In vivo: sciatic nerve of adult female Lewis rats	poly(l-lactide-co-caprolactone), poly(propylene glycol) and sodium acetate	Electrospinning	The alignment of scaffold fibers influenced the neural tissue regeneration, mechanical properties of NGC, and the amplitude and conduction velocity of compound muscle action potential (CMAP).
Subramanian et al., 2011 [44]	In vitro: SC	Poly(lactide-co-glycolide) (PLGA)	Electrospinning	The alignment of the scaffold fibers influenced biodegradation and porosity rate, tensile strength, and longitudinal elasticity. This alignment assisted the direction of SC and influenced its proliferation.
Cooper et al., 2011 [45]	In vitro: SC; PC-12 cells	Chitosan–polycaprolactone (chitosan–PCL)	Electrospinning	Alignment of the scaffold fibers influenced the environment for nerve cell proliferation, neurite extension, phenotype, and morphology cell. Additionally, elicit chemical and topographical cues for the modulation of neuritogenesis.
Wang et al., 2011 [46]	In vitro: DRG from SD rats	Poly(propylene carbonate) (PPC)	Electrospinning	Alignment of the scaffold fibers determines growth orientation neurite and SC migration. Additionally, alignment affected the speed of neurite growth and distances of SC migration.
Neal et al., 2012 [47]	In vitro: PC-12 cells, DRG In vivo: Rat tibial nerve	Laminin and laminin–polycaprolactone (PCL)	Electrospinning	The alignment of scaffold fibers influenced retrograde nerve conduction speed, motor, and sensory function.
Kijeńska et al., 2012 [48]	In vitro: C17.2 nerve stem cells	Poly(l-lactic acid)-co-poly(ε-caprolactone) or P(LLA-CL), collagen I and collagen III	Electrospinning	Aligned scaffold fibers affected the average tensile strength, cell growth, and cell proliferation.
Masaeli et al., 2013 [49]	In vitro: SC	Poly (3-hydroxy-butyrate) (PHB) & Poly (3-hydroxy butyrate-co-3- hydroxyvalerate) (PHBV)	Electrospinning	Aligned scaffold fibers affected average tensile strength and elongation percentage nanofiber scaffolds. Additionally, the morphology of SC oriented along the fiber direction was affected.
Ouyang et al., 2013 [50]	In vitro: SC of rat	Collagen type I and poly(lactic-co-glycolic acid) (PLGA)	Electrospinning	Aligned scaffold fibers favored motor function, nerve conduction, markers expressions, SC morphology, axon regeneration, and myelination.
Xie et al., 2014 [51]	In vitro: DRG, SCin vivo: Sciatic nerve of rats	Poly(ε-caprolactone) (PCL)	Electrospinning	Bilayer NGC (random and aligned nanofibers layers) are more tear-resistant in surgical procedures due to isotropic mechanical properties. It was unclear if random nanofibers could interfere with the aligned nanofibers in the extension pattern of the neurites and interfere in nerve regeneration.
Radhakrishnan et al., 2015 [52]	In vitro: SC	Poly(lactide-*co*-glycolide)	Electrospinning	Aligned scaffold fibers favored the adhesion, proliferation, and morphology of cells. Additionally, the expression of myelination markers and maturation of SC was regulated.
Yan et al., 2015 [53]	In vivo: Sciatic nerve of adult SD rats	Poly(l-lactic acid-*co*-e-caprolactone)	Electrospinning	Alignment of the scaffold fibers influenced cell growth orientation, myelination, and neuropathic pain post-injury.
Gnavi et al., 2015 [54]	In vitro: Primary cultures, SC line RT4-D6P2T, and cell line 50B11.	Gelatin	Electrospinning	The alignment of the scaffold fibers influenced adhesion and proliferation cell.
Panahi-Joo et al., 2016 [10]	In vitro: PC12, SH-SY5Y and C6 cell culture	Polycaprolactone (PCL)	Electrospinning dual pole: fibers alignedConventional electro-spinning: semi-aligned and random fibers.	The alignment of the scaffold fibers influenced topographical guidance, growth, elongation, and migration of cells. The tensile strength and degree of crystallinity of the fibers were also influenced.
Ranjbar-Mohammadi et al., 2016 [55]	In vitro: PC12 cells	Poly(l-lactic acid) and gum tragacanth (PLLA/GT 100:0, 75:25, 50:50).	Electrospinning	Aligned scaffold fibers favored cell orientation and elongation.
Zhang et al., 2018 [56]	In vivo: Sciatic nerve, Adult Sprague–Dawley rats In vitro: SC	Poly(3-hydroxybutyrate-co-3-hydroxyvalerate) (PHBV) & polyethylene oxide (PEO): PHBVPEO	Electrospinning	The alignment of the scaffold fibers influenced cell orientation and elongation.
Gnavi et al., 2018 [57]	In vitro: RT4-D6P2T SC line and DRG neuronal 50B11 cell line cultures and DRG explants	Gelatin (GL) and Chitosan (CTS)	Electrospinning	Aligned scaffold fibers favored cell adhesion and proliferation rates and filopodia formation.
Karimi et al., 2018 [58]	Pre-Experimental Study of BD	Poly(3-hydroxybutyrate) (PHB) solutions with chitosan (15%, 20%)	Electrospinning	The alignment of the scaffold fibers influenced scaffold morphology, hydrophilicity, and mechanical properties.
Quan et al., 2019 [6]	In vitro: SC, PC12 cells and DRG In vivo: Sciatic nerve of adult female rats	Polycaprolactone and 2% chitosan	Electrospinning	The alignment of the scaffold fibers influenced the expression of ATF3 and caspase-3 at the regenerative matrix and distal nerve segment. Thus, influencing nerve function, muscle mass, and recovery of distal nerve ultrastructure.
Yachao Jia et al., 2019 [59]	In vivo: Rat sciatic nerve In vitro: SC line RSC96	Poly(l-lactic acid-co-ε-caprolactone)	Electrospinning	Aligned scaffold fibers influenced macrophage elongation along the fibers, and induced the development of M2 macrophage type (pro-healing) or pro-inflammatory M1 type. Additionally, the proliferation and migration of SC were affected.
Zhang et al., 2020 [60]	In vitro: RSC96 and PC-12 cell lines In vivo: Sciatic nerve of rats	Poly(l-lactic acid) (PLLA)/soy protein isolate (SPI)	Electrospinning	Alignment of the scaffold fibers was determinant in the regeneration and functional reconstruction of the injured nerve.

**Table 6:** Biodegradability of an NGC with the different morphological characteristics. ↑ and ↓ means increasng and deacrising.

↑ Fiber diameter	↓ Biodegradability
↑ Porosity	↑ Biodegradability
↑ Pore size	↑ Biodegradability
Random Fibers	↑ Biodegradability

## Data Availability

Data sharing not applicable.

## Abbreviations

NGC	Nerve Guide Conduits
ECM	Extracellular matrix
CNS	Central Nervous System
SCs	Schwann cells
UTS	Ultimate Tensile Strength
EM	Elastic modulus
EB	Elongation at break
NTFs	Neurotrophic factors
